# The RNA-Binding Protein DDX18 Promotes Gastric Cancer by Affecting the Maturation of MicroRNA-21

**DOI:** 10.3389/fonc.2020.598238

**Published:** 2021-01-08

**Authors:** Yeqian Zhang, Fengrong Yu, Bo Ni, Qing Li, Seong-Woo Bae, Jong-Ho Choi, Han-Kwang Yang, Seong-Ho Kong, Chunchao Zhu

**Affiliations:** ^1^ Department of Gastrointestinal Surgery, Renji Hospital, School of Medicine, Shanghai Jiao Tong University, Shanghai, China; ^2^ State Key Laboratory of Oncogenes and Related Genes, Shanghai Cancer Institute, Renji Hospital, School of Medicine, Shanghai Jiao Tong University, Shanghai, China; ^3^ Department of Surgery, Seoul National University Hospital, Seoul, South Korea; ^4^ CancerResearch Institute, Seoul National University, Seoul, South Korea

**Keywords:** gastric cacner, ncRNA (noncoding RNA), DEAD box family, PTEN (phosphatase and tensin homolog deleted on chromosome 10), Drosha-independent miRNA

## Abstract

**Objectives:**

The noncoding RNAs (ncRNAs) play important roles in gastric cancer. Most studies have focused on the functions and influence of ncRNAs, but seldom on their maturation. DEAD box genes are a family of RNA-binding proteins that may influence the development of ncRNAs, which attracted our attention. By combining a small sample for high-throughput gene microarray screening with large samples of The Cancer Genome Atlas (TCGA) data and our cohort, we aimed to find some gastric cancer-related genes. We evaluated the clinical significance and prognostic value of candidate gene DDX18, which is overexpressed in gastric cancer tissues. To provide a theoretical basis for the development of new therapeutic targets for the treatment of gastric cancer, we investigated its effect on the malignant biological behavior of gastric cancer *in vitro* and *in vivo*, and also discuss its mechanism of action.

**Methods:**

(i) The differential profiling of mRNA expression in five pairs of gastric cancer and adjacent normal tissues was studied by Arraystar Human mRNA Microarray. By combining this with TCGA data and our cohort, we finally filtered out DDX18, which was upregulated in gastric cancer tissues, for further investigation. (ii) The protein expression of DDX18 was detected by immunohistochemistry staining. Then the relationship between the DDX18 expression level and the clinicopathological data and prognosis was analyzed. (iii) A CCK-8 assay and colony formation assay were used to evaluate the effect of DDX18 on cell growth and proliferation *in vitro*. A transwell assay was also performed to examine the migration and invasion of gastric cancer cells. Cell apoptosis was analyzed by using a fluorescein isothiocyanate–annexin V/propidium iodide double-staining assay. To identify the role of DDX18 in the tumorigenic ability of gastric cancer cells *in vivo*, we also established a subcutaneous gastric cancer xenograft model. Coimmunoprecipitation, small RNAseq, and western blotting were performed to explore the mechanism of action of DDX18 in gastric cancer. A patient-derived xenograft (PDX) model was used to confirm the effect of DDX18 in gastric cancer tissues.

**Result:**

(i) DDX18 was upregulated in gastric cancer tumor tissues from a TCGA database and our cohort. The expression of DDX18 was also closely related to tumor volume, Borrmann classification, degree of tumor differentiation, cancer embolus, lymph node metastasis, and TNM stage. (ii) DDX18 could promote cell proliferation, migration, and invasion and inhibit cell apoptosis *in vivo* and *in vitro*. (iii) DDX18 could promote the maturation of microRNA-21 through direct interaction with Drosha, decreasing PTEN, which could upregulate the AKT signaling pathway. (iv) The PDX model showed that DDX18 could promote the proliferation of gastric cancer tissues by means of the PTEN–AKT signaling pathway.

**Conclusions:**

(i) DDX18 can be treated as a molecular marker to assess the prognosis of patients with gastric cancer. (ii) DDX18 could be a potential therapeutic target in gastric cancer.

## Introduction

As the third most deadly cancer worldwide, gastric cancer is a serious threat to human health (1). While early gastric cancer has a good cure rate, advanced or late gastric cancer often has a poor prognosis. Therefore, it is very important to find specific biomarkers to evaluate and predict the occurrence and metastasis of gastric cancer and assess treatments to improve the diagnosis and treatment of this disease.

An increasing number of studies have shown that noncoding RNAs (ncRNAs) such as microRNAs and long noncoding RNAs (lncRNAs) play an important role in the occurrence and development of gastric cancer. However, these studies have mainly focused on the regulation and influence of microRNAs and lncRNAs on downstream genes. How ncRNAs are regulated is relatively unknown.

The DDX (DEAD-box gene) family is a family of RNA helicases that was first proposed by Patrick Linder in 1989 (2, 3). The DEAD box has nine conserved regions (Q, I, Ia, Ib, II–VI) and region II, which includes D (Asp), E (Glu), A (Ala), and D, which has been recognized as a characteristic sequence. These proteins are obtained by hydrolysis of ATP and then release RNAs to regulate cellular processes such as translation initiation, nuclear and mitochondrial splicing, and ribosome and splice packaging. Previous studies have suggested that the DDX family members are expressed abnormally in tumor tissues, which is related to tumor occurrence, development, metastasis, and invasion (4–6).

The expression levels of mature microRNAs are mainly determined by a critical rate-limiting step: the processing of premature microRNAs (pri-microRNAs and pre-microRNAs) (2). The Drosha microprocessor, whose core components are Drosha and DGCR8, functions first in microRNA maturation (7). Nevertheless, neither Drosha nor DGCR8 can recognize the indicated pri-microRNAs with any specificity (8). Regulatory components are needed in the microprocessor to offer specificity for recruiting and processing pri-microRNAs, accounting for the finding that the same primary transcript could generate diverse expression levels of mature microRNA.

In this study, we first used gene chip technology to explore the differentially expressed genes in gastric cancer. Further analysis of the clinical and pathological data identified the high expression of DDX18 in gastric cancer tissues. We performed cell function experiments to study the role of DDX18 in gastric cancer. We also explored its molecular mechanism by means of molecular cytology technology and animal experiments to elucidate DDX18 and to determine its role in the occurrence and development of gastric cancer.

## Materials and Methods

### Patients and Tissue Samples

Thirty-seven pairs of fresh gastric cancer and control normal gastric tissue specimens were obtained during surgery carried out on 37 patients from January 2013 to August 2014. All 37 patients underwent resection of primary gastric cancer at Renji Hospital, School of Medicine, Shanghai Jiao Tong University, Shanghai, China. Resected cancer tissues and paired noncancerous tissues were immediately cut and frozen in liquid nitrogen, and kept at –80°C until RNA and DNA extraction for quantitative real-time polymerase chain reaction (PCR).

For the assessment of immunoreactivity and the prognostic value of DDX18 in gastric cancers, the inclusion criteria for patients with gastric cancer were as follows: (i) a distinct pathological diagnosis of gastric adenocarcinoma; (ii) no radiotherapy, chemotherapy, or other anticancer therapies prior to surgery; (iii) primary tumor resection, including radical gastrectomy and palliative gastrectomy; and (iv) availability of complete clinicopathological and follow-up data.

A total of 585 paraffin-embedded tissue samples that met the above criteria were collected from patients with gastric cancer at the Department of Gastrointestinal Surgery, Renji Hospital, from January 2006 to December 2011 for tissue microarray construction and immunohistochemistry (IHC) staining. The overall survival (OS) is calculated from the date of tumor resection until death.

### Cell Lines and Cell Cultures

Cells from the human gastric cancer cell lines SGC-7901, NCI-N87, HGC27, MGC-803, BGC-823, and AGS were purchased from the Institute of Biochemistry and Cell Biology, Chinese Academy of Sciences, Shanghai, China. All the cells were cultured in Roswell Park Memorial Institute (RPMI) 1640 medium (Invitrogen, Carlsbad, CA, USA). The medium contained 10% fetal bovine serum and 1% penicillin/streptomycin. All cells were incubated in a cell incubator under 5% CO_2_ at 37°C.

### Lentivirus Transfection

Genomeditech (Shanghai, China) assisted in the design and production of DDX18 short hairpin RNA (shRNA).

Six-well plates were prepared and inoculated with the appropriate gastric cancer cells, with the adherent cells occupying about 50% of the total area of the plates. The appropriate amount of lentivirus was added to each well according to the multiplicity of infection value of gastric cancer cells. The cells were screened with antibiotics recommended by Genomeditech, and the transfection efficiency was measured by fluorescence quantitative PCR or western blot. The target sequences of DDX18 were: sh-1, sense: 5′–3′, GCAGCGGAACCTAAAGTTT; sh-2, sense: 5′–3′, GCATACCTATGGCTTGATA; sh-control, sense: 5′–3′, TTCTCCGAACGTGTCACGT.

### RNA Extraction and Quantitative Real-Time PCR

Total RNA was extracted with Trizol and reverse-transcribed into complementary DNA (cDNA) by PrimeScript™ (Takara Biomedical Technology, Beijing, China). Using glyceraldehyde 3-phosphate dehydrogenase (GAPDH) as the internal reference, real-time PCR analysis was conducted using an Applied Biosystems 7500 Real-Time PCR System (biological system), and the relative expression level of the target genes was calculated by the 2^–ΔΔCt^ method. The primer sequences were: DDX18: 5′–3′, F-ATGTCACACCTGCCGATGAAA, R-CCCTGAAACTTTAGGTTCCGC; GAPDH: 5′–3′, F-GGAGCGAGATCCCTCCAAAAT, R-GGCTGTTGTCATACTTCTCATGG.

### Western Blotting

Gastric cancer cells were lysed by radioimmunoprecipitation assay (RIPA) buffer (Beyotime, Beijing, China) and protease inhibitors (Roche, CA, USA). Then, 10% sodium dodecyl sulfate glue was used for electrophoresis and electrorotation to transfer the protein to the nitrocellulose (NC) membrane. The NC membrane was blocked for 1 hour at room temperature in Tris-buffered saline containing 5% skimmed milk. After incubation with primary and secondary antibodies, electrochemiluminescence was used to obtain bands (antibodies: DDX18 Abcam ab70527; P-AKT cell signaling SER473; AKT 10174-2-AP; PTEN Abcam ab32199).

### Coimmunoprecipitation

We linked three labels (FLAG-DDX18, HA-Drosha, His-DGCR8) to the indicated proteins. The prepared cells transfected with FLAG-DDX18, HA-Drosha, and His-DGCR8 plasmids were collected for nuclear protein extraction followed by coimmunoprecipitation. A nuclear protein extraction kit (P0027, Beyotime) was used according to the manufacturer’s protocol. In brief, alternate vortexing and centrifugation combined with the extraction kit were used to separate the total proteins into nuclear and cytoplasmic proteins. At the same time as the cell nuclear proteins were prepared, protein A/G magnetic beads (B23201; Bimake, Shanghai, China) were preincubated on a spinning wheel at 4°C for 30–60 minutes and washed three times with PBS. The antibody complex was then suspended in the nuclear protein solution. After the protein solution was fully combined with the magnetic bead–antibody complex, the extraction buffer was washed three times. Magnetic separation was performed by heating. The immunoprecipitate was collected and western blotting was performed.

### Cell Proliferation

The proliferation capacity of gastric cancer cells was determined by cell count kit 8 (CCK-8; Beyotime, China). After transfection with DDX18 shRNA, gastric cancer cells were inoculated in 96-well plates, with about 2000 cells per well. Under dark conditions, each chamber was incubated for 1 hour with 10 µl CCK-8 reagent and the optical density was measured at a wavelength of 450 nm with a SpectraMax Plus 384 (Molecular Devices). Six-well plates were used to lay about 500 gastric cancer cells in each well, and then the cells were allowed to grow for about 2 weeks. After washing twice with phosphate-buffered saline (PBS), cells were fixed in 4% paraformaldehyde for 15 minutes, and 0.5% crystal violet staining was performed for a further 15 minutes. The number of gastric cancer cell clones in different groups was calculated.

### Detection of Apoptosis

For the cell apoptosis assay, 20 × 10^5^ cells per well were cultured under serum deprivation in six-well plates. Adherent cells were detached with 0.25% trypsin without ethylenediaminetetraacetic acid in 1 × PBS. Cells were harvested in complete RPMI 1640 and centrifuged at 1000 r.p.m. for 5 minutes. Each batch of cells was washed with 1 × PBS and stained with 50 mg/ml propidium iodide (PI) and annexin V–fluorescein isothiocyanate according to the manufacturer’s instructions. The percentage of annexin V (+) and PI (–) cells was analyzed by flow cytometry.

### Transwell Assay

A transwell chamber was prepared with 600 µl serum-free RPMI 1640 medium added to each well, followed by 5 × 10^5^ cells. The culture medium containing 20% serum was used in the lower layer. After 24 hours, the chamber was washed twice with PBS, 4% paraformaldehyde was used to fix the cells for 15 minutes, crystal violet was used for staining for 15 minutes, and the number of cells passing through the chamber was counted under a microscope.

### Luciferase Reporter Gene

The DDX18 gene, PTEN gene 3′-untranslated region (UTR) target clone (CmiT010099-MT05), and negative control clone (CmiT000001-MT05) were purchased from GengCopia Company, USA. For the secretory double luciferase reporter gene analysis, 293T cells were inoculated in six-well plates and cotransfected when the cell confluence reached 50%. The culture medium was changed to whole medium 24 hours after transfection and incubation continued. The supernatant of the cell culture solution was carefully collected 48 hours after transfection and packed into two tubes, each containing 200 μL, which was used for the detection of Gaussia luciferase (GLuc) and secretory alkaline phosphatase (SeAP), respectively. The supernatant used to detect SeAP was placed in a water bath at 65°C for 15 minutes; the working solution of 1 × glucose was prepared and incubated in the dark at room temperature for 25 minutes; the working solution of 1 × SeAP was prepared and incubated in the dark at room temperature for 10 minutes; 10 µl of supernatant and 100 μl of the corresponding working solution were added to each well of a 96-well plate, and multiples of three were used in each group. After incubation at room temperature for 1–10 minutes, the fluorescence was determined using a microplate reader. For data processing, with the SeAP fluorescence reading as the internal reference, the ratio of the GLuc fluorescence reading to the SeAP fluorescence reading was compared among the groups, and the mean values of three reading plates and three complex holes were taken.

### Immunohistochemistry and Staining Evaluation

Paraffin sections were dewaxed with xylene, rehydrated by fractional ethanol, and antigens were extracted. The sections were blocked with 10% bovine serum albumin (BSA), incubated with primary antibody for 1 hour, and then incubated with secondary antibody for 30 minutes at room temperature. DAPI (4′,6-diamino-2-phenylindole hydrochloride; AppliChem, A4099) was used to stain the nucleus. An automatic fluorescence microscope (Nikon) was used for image observation and analysis. The tissue sections were assessed and graded by two independent investigators who were unaware of the clinicopathological factors. The staining intensity was 0 (negative), 1 (weak), 2 (medium), and 3 (strong).The degree of staining was stratified as 0 (0%), 1 (1–25%), 2 (26–50%), 3 (51–75%), and 4 (76–100%), defined as the percentage of positive staining area in the total tumor invasion area. The final score of DDX18 expression was 0–7. The samples were divided into two groups: low DDX18 expression (0–3 points; IHC-0) and high DDX18 expression (4–7 points; IHC-1,2).

### Cellular Immunofluorescence

After immersion in 100% alcohol, alcohol lamp burning, and ultraviolet irradiation, a glass slide was placed into a six-well plate, and gastric cancer cells were spread on the glass slide. The cells were allowed to grow to 30–50% of the area of the glass slide on the second day. Cells were fixed with 4% paraformaldehyde for 15 minutes and treated with 0.5% Triton X-100 for 1 minute. Nonspecific binding sites were blocked by 1% BSA. The primary antibody was incubated for 1 hour at room temperature and the secondary antibody for 30 minutes, and then treated with DAPI for 30 minutes for nuclear staining. Observation and analysis were conducted by automatic fluorescence microscope (Nikon).

### Animal Models

To construct the subcutaneous tumor model of nude mice, 5 × 10^6^ gastric cancer cells were injected into the left axilla of each nude mouse. After 4 weeks, the nude mice were killed, the subcutaneous tumor was removed, and its weight and volume were measured; 4% paraformaldehyde was used to store the tumor. All animal experiments were approved by the Ethics Committee of Renji Hospital.

### Statistical Analysis

SPSS 22.0 (SPSS Inc., Chicago, IL, USA) was used to analyze and calculate all the data, and the value was the mean ± SD. Student’s *t*-test and the chi-squared test were used in the study. The Cox proportional hazard model was used for univariate and multivariate analysis to understand the factors affecting survival. *P* < 0.05 was considered statistically significant.

## Results

### DDX18 Is Outstanding in Gastric Cancer Gene Chip Analysis

**Figure 1:**
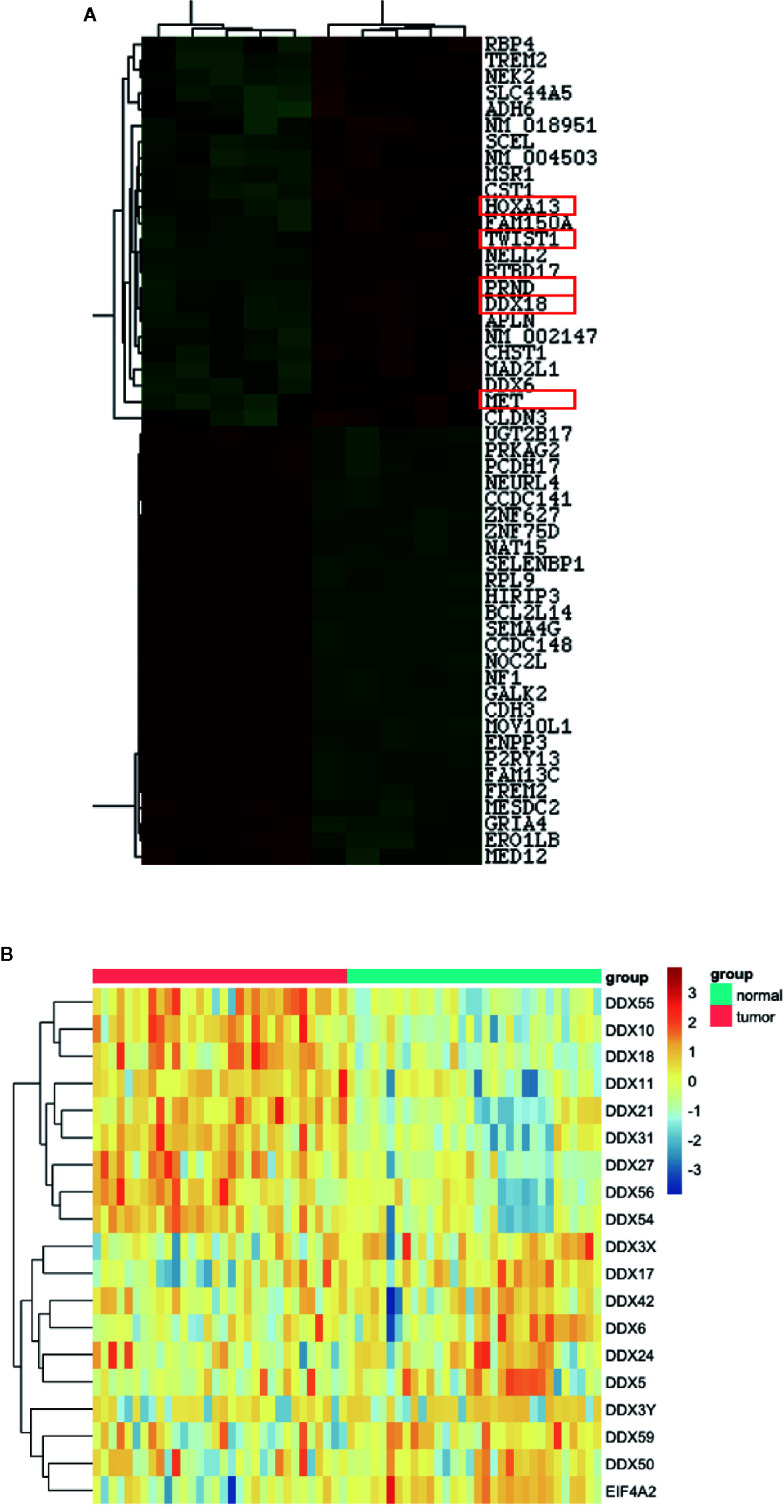
DDX18 is highly expressed in gastric cancer. **(A)** DDX18 expression on a gene chip. **(B)** DDX gene family expressions in The Cancer Genome Atlas (TCGA) database.

The mRNA expression profile of the known protein in five cases of gastric cancer and adjacent tissues was analyzed by an Arraystar Human mRNA Microarray v2.0 gene chip. The GAPDH gene was used as the internal control, and the fold difference between the different groups of >2 or <–2 with *P* < 0.05 was used for standard screening. In all the genes upregulated, five genes with the highest fold change were obtained, which were TWIST2, MET, HOXA13, DDX18, and PRND (**Figure 1A**).

### Differential Expression of DDX18 in gastric cancer tissues

**Figure 2:**
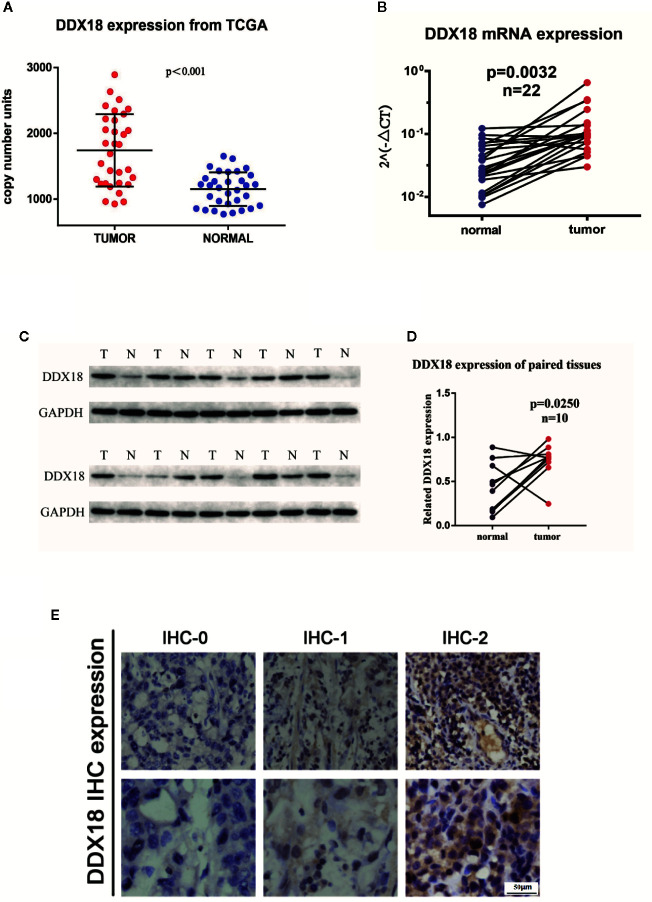
DDX18 expressions in gastric cancer tissues. **(A)** DDX18 expressions from The Cancer Genome Atlas (TCGA). **(B)** DDX18 mRNA expression in 22 paired gastric cancer tissues. **(C, D)** DDX18 protein expressions in 10 paired gastric cancer tissues. **(E)** DDX18 expression by immunohistochemistry.

First, we assessed five pairs of gastric cancer tissues and matched paracancerous tissues by using gastric cancer gene chip technology. We used a fold change >2 and *P* < 0.05 as the inclusion criteria, and DDX18 attracted our attention among the top differentially expressed genes (**Figure 1A**). In other words, the DDX family of genes caught our attention. Combining this with The Cancer Genome Atlas (TCGA) database, we focused on DDX18 as our target of interest (**Figure 1B**). Next, we examined DDX18 in 22 pairs of fresh gastric cancer tissues from a large sample set by quantitative PCR (**Figure 2B**) and western blot analyses (**FigureS 2C, D**), which confirmed that DDX18 was specifically highly expressed in gastric cancer tissues (*P* < 0.05). Similar results were verified in the TCGA database (**Figure 2A**).

Next, we detected the expression of DDX18 in 585 cases of gastric cancer by IHC. IHC-0 indicated negative DDX18 expression, and IHC-1 and IHC-2 indicated positive DDX18 expression, according to immunohistochemical grade (**Figure 2E**). Subsequently, we analyzed the relationship between the DDX18 protein level and the clinicopathological features of gastric cancer in 585 cases of gastric cancer. The results of univariate analysis showed that positive DDX18 protein expression was present in 65.0% (380/585) of gastric cancer cases. The expression level of DDX18 was closely related to tumor location, tumor size, Borrmann classification, differentiation, intravascular tumor thrombus formation, nerve invasion, depth of invasion, lymph node metastasis, and TNM staging but not to age or gender.

**Figure 3:**
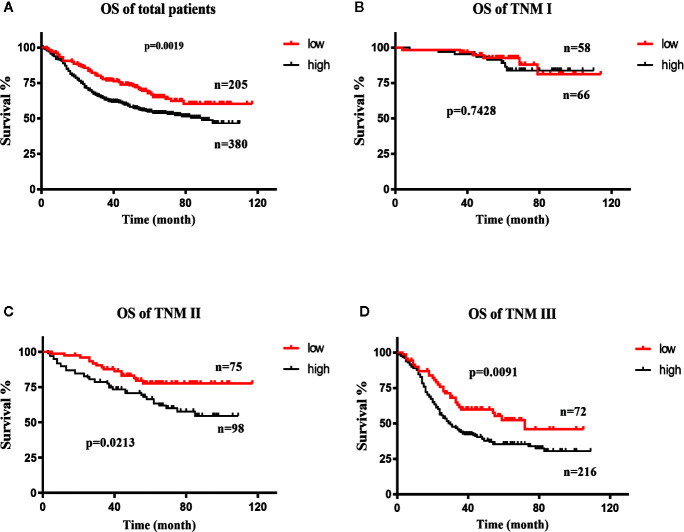
Survival curve of DDX18. **(A)** Overall survival (OS) curve of DDX18. **(B)** Survival curve of patients with TNM stage (I) **(C)** Survival curve of patients with TNM stage II. **(D)** Survival curve of patients with TNM stage III.

All patients were divided into the DDX18 high-expression group (a total of 380 patients with IHC-1 and IHC-2) and the DDX18 low-expression group (a total of 205 patients with IHC-0), which were divided by DDX18 immunohistochemistry. The OS rates of the patients in the low-expression group were 90.6%, 76.7%, and 67.8%. The OS rates of the patients with high expression were 88.9%, 63.1%, and 54.7%. The difference between the two groups in the first stage of gastric cancer was not significant, but in stage II and stage III gastric cancer significant differences were observed (**Figures 3A–D**).

The results showed that the degree of tumor differentiation, lesion site, Borrmann classification, tumor size, intravascular thrombosis, nerve invasion, depth of invasion (T stage), lymph node metastasis (N stage), and DDX18 expression level had significant effects on the OS rate of 585 patients with gastric cancer, and the difference was significant (*P* < 0.05) (**Table 1**). The depth of invasion (T stage), lymph node metastasis (N stage), and DDX18 expression were independent prognostic risk factors (**Tables 2** and **3**).

**Table 1:** Association of DDX18 expression with clinicopathological characteristics in 585 patients with gastric cancer.

Clinicopathological characteristic	No. of cases	DDX18 expression				χ^2^	*P*-value
		Negative		Positive			
		*n* = 205 (35.0%)		*n* = 380 (65.0%)			
**Gender**							
Male	400	139	34.8%	261	65.3%	0.048	0.827
Female	185	66	35.7%	119	64.3%		
**Age (years)**							
≤60	273	94	34.4%	179	65.6%	0.084	0.772
>60	312	111	35.6%	201	64.4%		
**Tumor location**							
Upper	76	22	28.9%	54	71.1%	967.9	<0.001***
Middle	115	36	31.3%	79	68.7%		
Lower	309	116	37.5%	193	62.5%		
Whole	80	29	36.3%	51	63.8%		
Residual	5	2	40.0%	3	60.0%		
**Tumor size (cm)**							
<5	307	124	40.4%	183	59.6%	8.118	0.004**
≥5	278	81	29.1%	197	70.9%		
**Borrmann** ^a^							
I	33	13	39.4%	20	60.6%	509.1	<0.001***
II	76	29	38.2%	47	61.8%		
III	333	111	33.0%	223	67.0%		
IV	66	18	27.3%	48	72.7%		
**Differentiation**							
High	15	8	53.3%	7	46.7%	248.5	<0.001***
Medium	146	63	43.2%	83	56.8%		
Low	424	134	31.6%	290	68.4%		
**Vessel invasion**							
Negative	486	182	37.4%	304	62.6%	7.302	0.007**
Positive	99	23	23.2%	76	76.8%		
**Nerve invasion**							
Negative	504	188	37.3%	316	62.7%	8.159	0.004**
Positive	81	17	21.0%	64	79.0%		
**T stage**							
T1	76	34	44.7%	42	55.3%	586.7	<0.001***
T2	85	39	45.9%	46	54.1%		
T3	151	52	34.4%	99	65.6%		
T4	273	80	29.3%	193	70.7%		
**N stage**							
N0	228	106	46.5%	122	53.5%	596.6	<0.001***
N1	105	35	33.3%	70	66.7%		
N2	113	25	22.1%	88	77.9%		
N3	139	39	28.1%	100	71.9%		
**TNM stage**							
I	124	58	46.8%	66	53.2%	370.8	<0.001***
II	173	75	43.4%	98	56.6%		
III	288	72	25.0%	216	75.0%		

a Borrmann for 509 cases of advanced gastric cancer. *P < 0.05, **P < 0.01, ***P < 0001.

**Table 2:** Univariate analysis of overall survival in 585 patients with gastric cancer.

Univariate analysis			
Clinicopathology	Hazard ratio	95% confidence interval	*P*-value
Age	0.853	0.656–1.108	0.234
Gender	1.234	0.967–1.589	0.089
Differentiation	1.674	1.233–2.273	0.001
Tumor location	1.535	1.083–2.176	0.016
Borrmann	1.7	1.267–2.281	<0.001
Tumor size	1.578	1.197–2.081	0.001
Nerve invasion	1.933	1.447–2.734	<0.001
Vessel invasion	2.198	1.640–2.946	<0.001
T stage (1, 2/3, 4)	0.252	0.170–0.373	<0.001
N stage (0, 1/2, 3)	0.242	0.185–0.315	<0.001
TNM stage (1 + 2/3)	0.237	0.178–0.314	<0.001
DDX18 expression	2.605	2.208–3.345	<0.001

**Table 3:** Multivariate Cox regression analysis of overall survival in 585 patients with gastric cancer.

Multivariate analysis			
Clinicopathology	Hazard ratio	95% confidence interval	*P*-value
Differentiation	1.12	0.815–1.538	0.484
Tumor location	1.132	0.856–1.498	0.384
Borrmann	1.02	0.746–1.349	0.903
Tumor size	1.176	0.887–1.559	0.26
Nerve invasion	1.203	0.850–1.701	0.297
Vessel invasion	1.145	0.828–1.582	0.413
T stage (1, 2/3, 4)	0.607	0.366–1.006	0.049
N stage (0, 1/2, 3)	0.453	0.296–0.694	<0.001
TNM stage(1 + 2/3)	0.411	0.306–0.533	<0.001
DDX18 expression	1.513	1.150–1.991	0.003

### Impact of DDX18 on Gastric Cancer Cell Lines

**Figure 4:**
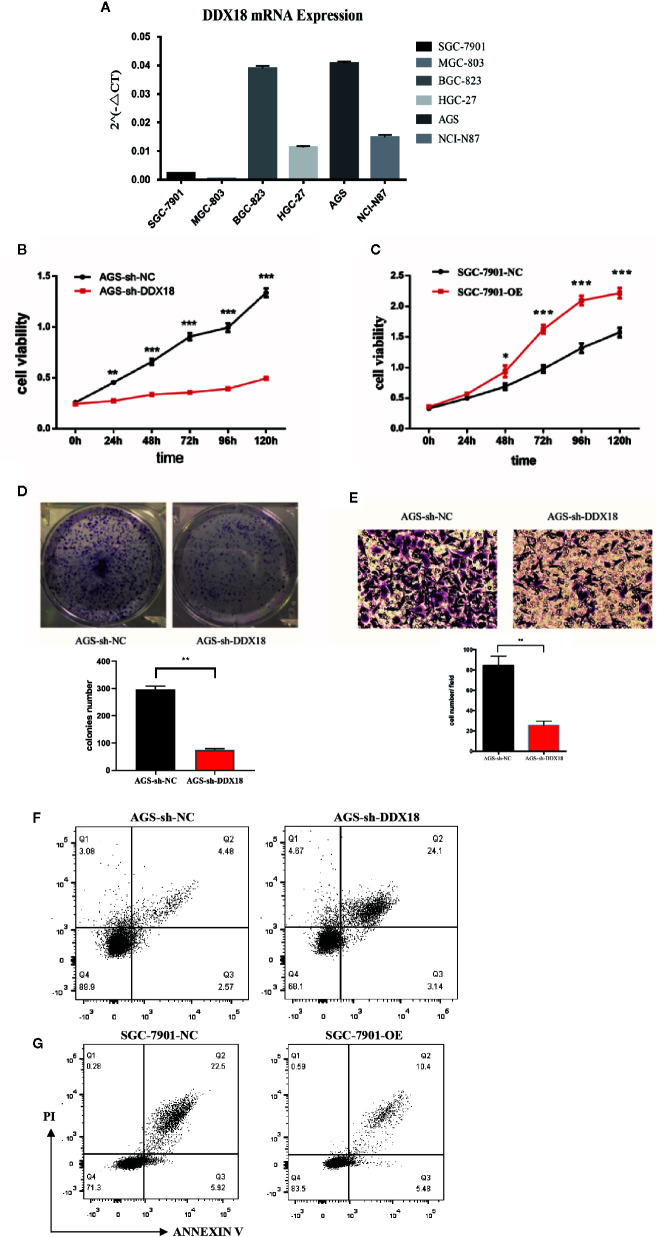
Impact of DDX18 on gastric cancer cell lines. **(A)** DDX18 expression in gastric cancer cell lines. **(B)** DDX18 knockdown decreased the proliferation of gastric cancer cells. **(C)** DDX18 overexpression promotes the proliferation of gastric cancer cells. **(D)** DDX18 knockdown decreases clonal formation of gastric cancer cells. **(E)** DDX18 knockdown decreases invasion of gastric cancer cells. **(F)** DDX18 knockdown increases the apoptotic rate of gastric cancer cells. PI, propidium iodide. **(G)** DDX18 overexpression decreases the apoptotic rate of gastric cancer cells. PI, propidium iodide.

We detected the expression of DDX18 in gastric cancer cell lines (**Figure 4A**). Then we designed two target sequences of shDDX18, and a mixture of two shRNAs was used in the following experiments. The proliferative activity of the AGS-sh-DDX18 cells and the control cells was compared by the CCK-8 method. The OD_450_ values of each group were detected for five consecutive days. After 5 days, DDX18 knockdown significantly decreased the proliferation rate of tumor cells by 63.0% (*P* < 0.001) (**Figure 4B**). Subsequently, we compared the proliferative activity of the SGC-7901-OE cells and the control cells and found that exogenous DDX18 could significantly promote the growth of the SGC-7901 cells. The difference was significant (*P* < 0.001) (**Figure 4C**).

We found that the interference group’s ability to form clones (82.3 ± 5.31) was significantly lower than that of the control group (289.4 ± 11.2) (*P* < 0.01) (**Figure 4D**). The results demonstrated that DDX18 has a significant effect on the proliferation of individual adherent cells.

The transwell results showed that the number of penetrating cells in the control group was 83.6 ± 12.5 cells/high-power field (HPF), which was significantly higher than that of the interference group (26.6 ± 4.3 cells/HPF). The migration of the AGS cells was significantly weaker than that of the control group (*P* < 0.01) after stable knockdown of DDX18 gene expression (**Figure 4E**). Similar results were found in the BGC-823 cell line, which was another gastric cell line with high DDX18 expression (**Figure S1**).

In the cell apoptosis experiment, we used serum-free culture to induce apoptosis to study the effects of DDX18 on the absence of a nutrient supply. This enabled us to study the antiapoptotic ability.

The results showed that the average apoptotic rate of the interference group was 27.58 ± 3.63% compared with 7.03 ± 1.44% for the control group. The proportion of apoptotic cells increased significantly (*P* < 0.01), suggesting that interference with the DDX18 gene in the AGS cells significantly reduced the antiapoptotic ability (**Figure 4F**). Similar results showed that the average apoptosis rate of the overexpression group was 16.09 ± 2.24% after 48 hours of starvation, which was significantly lower than that of the control group (28.64 ± 4.33%, *P* < 0.01), suggesting that the antiapoptotic ability of the SGC-7901 cells was significantly enhanced after overexpression of the DDX18 gene (**Figure 4G**).

### Interaction of DDX18 and Drosha

**Figure 5:**
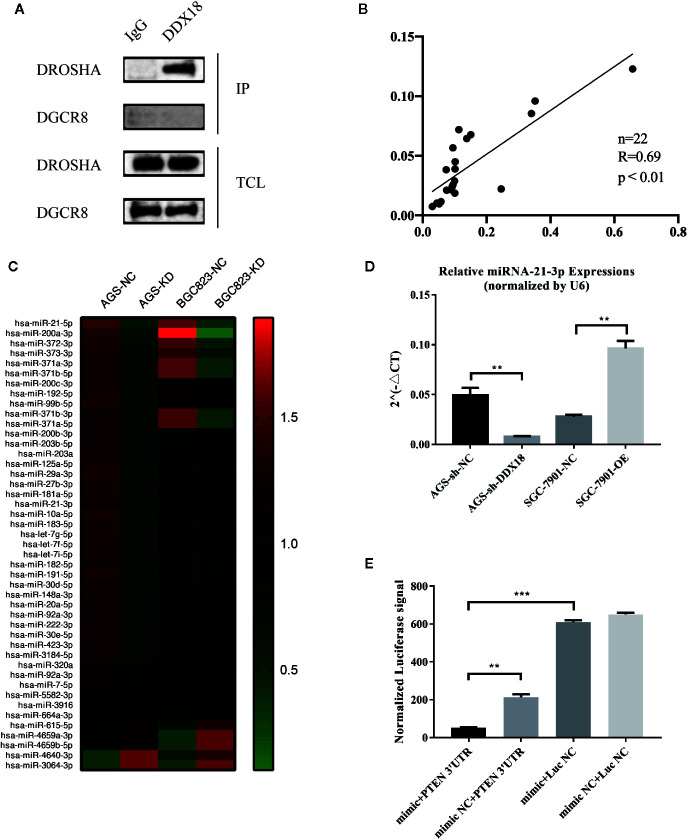
Interaction of DDX18 and Drosha. **(A)** DDX18 interacts with Drosha but not DGCR8. **(B)** The expression of microRNA-21 is correlated with that of DDX18. **(C)** MicroRNA expressions in the DDX18-KD cells. **(D)** DDX18 could affect the expression of microRNA-21. **(E)** MicroRNA-21 could bind to the 3′-untranslated region of PTEN and lead to degradation.

In a previous study, we found that DDX18 is highly expressed in gastric cancer. Cell and animal experiments have confirmed that DDX18 promotes the proliferation of gastric cancer cells. However, the mechanism that DDX18 exerted in this process remains unclear: DDX families belong to a family of RNA helicases with conserved DEAD domains, which have been found to play a role in various tumors. In addition to the original functions of DDX18 and the DDX family, we speculated that DDX18 might be related to the formation and maturation of RNAs. Therefore, we hypothesized that DDX18 might be related to the maturation of microRNAs. Next, we constructed a DDX18-KD cell model and detected the expression of microRNAs after DDX18 knockdown by small RNA-seq. We found that the expression of microRNA-21 in the DDX18-KD cell lines decreased significantly (**Figure 5C**). Combined with the detection of 22 clinical samples, the results showed that the expression of microRNA-21 was positively correlated with DDX18 expression (**Figure 5B**). Therefore, the results indicated that DDX18 can promote the expression of microRNA-21.

A common pathway exists for the maturation of microRNAs, i.e., pre-microRNAs sheared and matured through the action of Drosha and DGCR8. However, the expression of microRNAs differed in different cells. Therefore, there must be molecules that can specifically recognize and bind pre-microRNAs and affect their expression levels. Combined with the above research, we found a relationship between DDX18 and microRNA-21. Next, through coimmunoprecipitation experiments, we found that DDX18 could interact with Drosha but could not interact with DGCR8 (**Figure 5A**). Therefore, we propose that DDX18 could affect the expression of microRNA-21 by interacting with Drosha (**Figure 5D**).

### Confirmation of the DDX18→microRNA-21→PTEN/AKT Pathway

In a previous study, we identified the role of DDX18 in gastric cancer cells and confirmed that DDX18 could promote the expression of microRNA-21. Therefore, the mechanism of action of microRNA-21 in gastric cancer cells was examined next. By analyzing the sequence of microRNA-21, we speculated that microRNA-21 might bind to the 3′-UTR of PTEN, promoting the degradation of PTEN mRNA and affecting related pathways downstream.

To further confirm the direct effect of miR-21-3p and PTEN, we used the dual luciferase gene reporter system to verify that PTEN was the target gene for miR-21-3p. The following plasmids were cotransfected into 293T cells: mimic miR-21-3p + PTEN 3′-UTR target clone plasmid, mimic NC + PTEN 3′-UTR target cloning plasmid, mimic miR-21-3p + negative control fluorescein enzyme plasmid, and mimic NC + negative control luciferase plasmid. The results showed that the miR-21-3p mimic could significantly inhibit the activity of luciferase (*P* < 0.01) and confirmed the direct effect of miR-21-3p and the PTEN 3′-UTR (**Figure 5E**).

**Figure 6:**
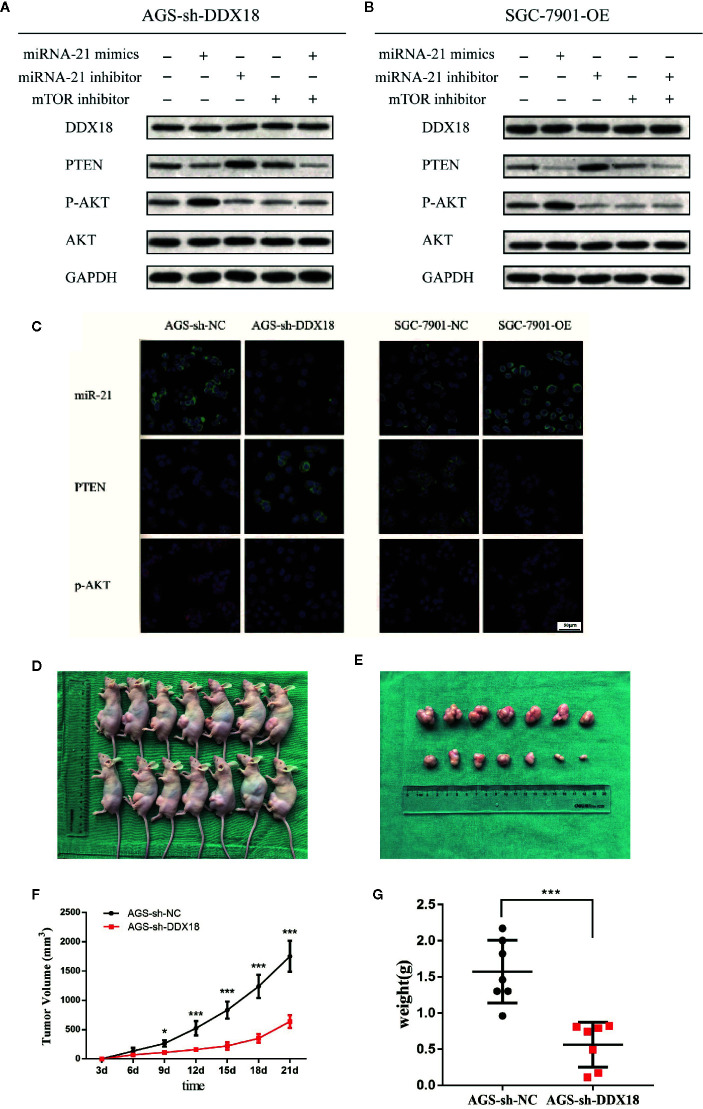
The DDX18→microRNA-21→PTEN/AKT pathway. **(A, B)** DDX18 affects the PTEN/AKT signaling pathway by controlling the maturation of microRNA-21. **(C)** DDX18 can also affect the expression of PTEN by regulating microRNA-21, which in turn affects the phosphorylation of AKT and regulates the AKT signaling pathway. **(D–G)** DDX18 interference strongly inhibited the AGS cell subcutaneous tumorigenic ability *in vivo*. *P < 0.05, ***P < 0001.

We performed western blotting to confirm that DDX18 affects the PTEN/AKT signaling pathway by controlling the maturation of microRNA-21. We added microRNA-21 mimics, microRNA-21 inhibitor, and mTOR inhibitor to the AGS-sh-DDX18 cell line and finally added microRNA-21 mimics and mTOR inhibitor to detect the expression levels of DDX18, PTEN, p-AKT, and total AKT. The results showed that the expression of PTEN was inhibited again after readministration of the microRNA-21 mimics in the AGS cell line, and the phosphorylation of AKT was enhanced (**Figure 6A**).

Subsequently, we added microRNA-21 mimics, microRNA-21 inhibitor, and mTOR inhibitor to the SGC-7901-OE cell line that had been transfected with DDX18 and finally added microRNA-21 inhibitor and mTOR inhibitor to detect the expression levels of DDX18, PTEN, p-AKT, and total AKT (**Figure 6B**). The results showed that the inhibition of PTEN was blocked by the addition of the microRNA-21 inhibitor, and the phosphorylation level of downstream AKT was significantly decreased. Therefore, DDX18 can indeed affect the expression of PTEN by regulating microRNA-21, which in turn affects the phosphorylation of AKT and regulates the AKT signaling pathway. This result confirms our hypothesis (**Figure 6C**).

### Effect of DDX18 on Tumor Formation *in Vitro*

AGS-sh-DDX18 cells and control AGS-sh-NC cells were used to induce subcutaneous tumor formation in the two groups of nude mice for 3 weeks. None of the mice in the two groups died during the experimental period. After 3 weeks of observation, the tumor-bearing nude mice were sacrificed, and the subcutaneous tumors were dissected. When the transplanted tumor was peeled from the skin, the tumor surface had a complete envelope with a clear boundary and slight adhesion to the surrounding tissue. We found that the average weight of the subcutaneous tumors in the control group was 1.57 ± 0.56 g, which was significantly different from that in the intervention group (*P* = 0.0003) and the interference group (0.56 ± 0.31 g). DDX18 interference strongly inhibited the AGS cell subcutaneous tumorigenic ability *in vivo*, which was consistent with the results of the *in vitro* experiments (**Figures 6D–G**).

**Figure 7:**
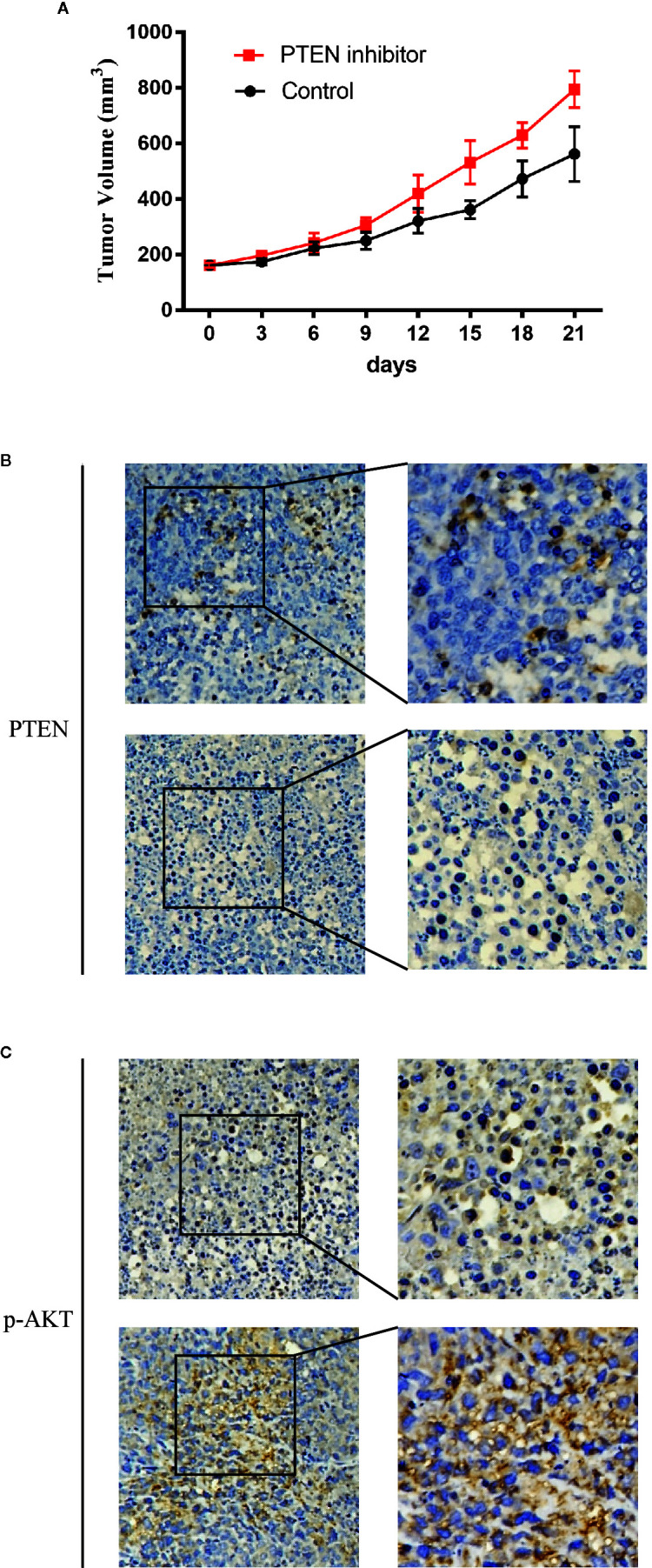
The effect of DDX18 on gastric cancer patient-derived xenograft (PDX) models. **(A)** The growth curve of DDX18 on PDX models by injection of SF1670. **(B, C)** Immunohistochemistry of the effect on PDX models by injection of SF1670.

Then we detected the influence of DDX18 on the patient-derived xenograft (PDX) models. PDX models with low DDX18 expression were implanted in the mice. From the third day, group 1 was injected with PBS every day, while the corresponding group 2 was injected with the PTEN inhibitor SF1670. After 21 days, we found that the injection of SH1670 could effectively promote tumor growth (**Figure 7A**). IHC detection showed that, with the injection of SF1670, PTEN expression was significantly decreased, while the expression of P-AKT was reactivated (**Figures 7B, C**).

## Discussion

Gastric cancer is one of the most common malignant tumors worldwide. Data from the World Health Organization have shown that the number of new cases of gastric cancer ranked fifth among all malignant tumors, following lung cancer, breast cancer, colorectal cancer, and prostate cancer (1). In addition to the high incidence of gastric cancer, the mortality rate of gastric cancer ranks third among all malignant tumors worldwide because of its high degree of malignancy, hidden early symptoms, and relatively low sensitivity to radiotherapy and chemotherapy. Gastric cancer poses a serious threat to the lives and health of people worldwide. This disease is a major burden to patients and families and also consumes many public health resources. Therefore, further study of the relationship between the clinicopathological characteristics of gastric cancer and the prognosis of patients, exploration of effective treatment methods, and comprehensive improvements in the overall level of diagnosis and treatment of gastric cancer are urgently needed.

In our study, we first discussed the differential expression of DDX18 in gastric cancer tissues and adjacent tissues, identifying the specific high expression of DDX18 in the cancer samples. Combined with the analysis of clinical prognosis data, we found that DDX18 was positively correlated with the degree of malignancy of gastric cancer and could significantly affect the prognosis of patients with gastric cancer. Further analysis showed that DDX18 could be applied as an independent risk factor for the prognosis of gastric cancer.

The role of ncRNAs, represented by microRNAs, in tumors, especially in gastric cancer, has been extensively studied in recent years (9–13). MicroRNAs can promote the degradation of RNA by targeting the 3′-UTR of the corresponding RNA, which can be regulated at the post-transcriptional level (14). At present, the maturation process of microRNAs is relatively clear. Pre-microRNAs can be further matured through the shearing effect of Drosha and eventually become mature microRNAs (15). However, the regulation of the formation, maturation, and expression of microRNAs is still unknown. The RNA helicase family has RNA helicase activity. The protein family represented by the DDX family is an important part of this group.

Certain members of the DEAD-box helicase (DDX) family have been defined as cofactors to the Drosha complex, which controls the post-transcriptional maturation of a specific group of microRNAs. Drosha is an RNase III enzyme, which binds and cleaves double-stranded RNA without any sequence specificity (15). It is known that one nucleotide substitute of pri-microRNA in the stem–loop structure would inhibit Drosha-mediated modification, suggesting that the stem–loop structure should be critical for the enzymatic activity of Drosha (16). The Drosha-mediated processing depends on the unique complexity in the sequence or structure of microRNA, even though all pri-microRNAs share some common features on one or more stem–loop structure. Some RNA-binding proteins, such as DDX18, serve as good candidates to identify this specificity through their selective interaction with targeted pri-microRNAs. Furthermore, Drosha harbors no helicase activity compared with another RNase III endonuclease, Dicer, which is also involved in pre-microRNA processing (17). Taken together, potential interaction between DDX18 and Drosha not only confers microRNA-binding specificity but also assists in the generation of a desirable stem–loop structure for the indicated pri-microRNAs, followed by efficient cleavage.

In this study, we first found that DDX18 was associated with the malignancy of gastric cancer. Based on the clinical prognosis of patients, we confirmed that DDX18 is abnormally highly expressed in patients with advanced gastric cancer and is closely related to a poor prognosis. Therefore, we speculated that DDX18 may play an important role in the development and progression of gastric cancer. Furthermore, we performed *in vivo* and *in vitro* experiments to detect the function of DDX18 in gastric cancer. Our findings showed that DDX18 promoted tumor growth. Knockdown of DDX18 also substantially decreased gastric cancer cell migration and invasion. Further studies showed that DDX18 could regulate the maturation of microRNA-21 and then affect the function of the downstream PTEN/AKT signaling pathway. Both microRNA-21 and the PTEN/AKT signaling pathway have already been proved to play an important role in gastric cancer (18–20).

In a previous study on microRNAs, we focused on the downstream target genes and the functions of microRNAs but did not examine the upstream regulation of microRNA expression and maturation. This study provides a new direction for DDX family and microRNA research. Moreover, we provide a preliminary experimental basis and theoretical basis for the future study of DDX18 as a new target for the treatment of gastric cancer.

## Data Availability Statement

The original contributions presented in the study are included in the article/**Supplementary Material**. Further inquiries can be directed to the corresponding authors.

## Ethics Statement

The studies involving human participants were reviewed and approved by Ethics Committee of Renji Hospital, School of Medicine, Shanghai JiaoTong University. The patients/participants provided their written informed consent to participate in this study. The animal study was reviewed and approved by Ethics Committee of Renji Hospital, School of Medicine, Shanghai JiaoTong University.

## Author Contributions

CZ and S-HK designed the study. YZ, FY, QL, BN, S-WB, and J-HC performed the *in vitro* and *in vivo* experiments. YZ and FY wrote this manuscript. H-KY provided valuable suggestions for this research. YZ provided help of bioinformatics analysis. CZ provided the clinical samples and prognostic data. CZ and S-HK provided the funding that supported this research. All authors contributed to the article and approved the submitted version.

## Funding

This research was supported by grants from the National Natural Science Foundation of China (nos. 81602062 and 81811540415).

## Conflict of Interest

The authors declare that the research was conducted in the absence of any commercial or financial relationships that could be construed as a potential conflict of interest.

## Supplementary Material

Supplementary Figure 1 Impact of DDX18 on BGC-823 cell lines. **(A)** DDX18 knockdown decrease clonal formation of BGC-823 cell line. **(B)** DDX18 knockdown decrease invasion of BGC-823 cell line.
