# Supplementary figures and images for: The RNA-Binding Protein DDX18 Promotes Gastric Cancer by Affecting the Maturation of MicroRNA-21
Source: Front Oncol. 2021 Jan 8;10:598238. doi: 10.3389/fonc.2020.598238 (PMC7821424; doi:10.3389/fonc.2020.598238)

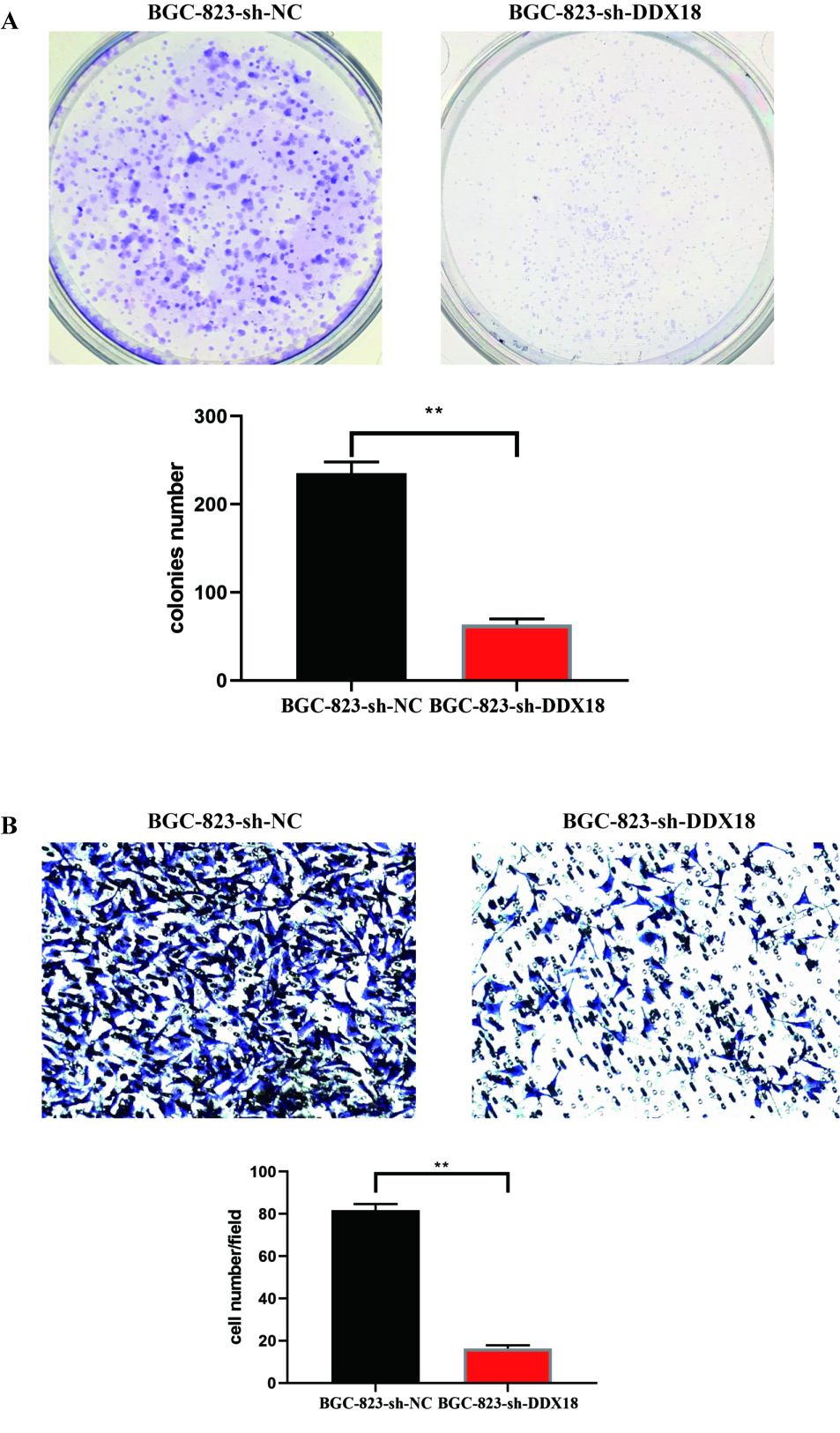

Supplement: Supplementary Figure 1 — Impact of DDX18 on BGC-823 cell lines. (A) DDX18 knockdown decrease clonal formation of BGC-823 cell line. (B) DDX18 knockdown decrease invasion of BGC-823 cell line. [file Image_1.tif]
